# Effect of Electrode Distances on Remediation of Eutrophic Water and Sediment by Sediment Microbial Fuel Cell Coupled Floating Beds

**DOI:** 10.3390/ijerph191610423

**Published:** 2022-08-21

**Authors:** Qing Wu, Jieqiong Liu, Qiannan Li, Wenjun Mo, Ruihan Wan, Sen Peng

**Affiliations:** School of Environmental Science and Engineering, Tianjin University, No. 135 Yaguan Road, Jinnan District, Tianjin 300350, China

**Keywords:** FB-SMFC, electrode distance, eutrophic water, synchronous remediation, bacteria diversity

## Abstract

Efficient and sustainable technologies for cleaning of contaminated water and sediments are in urgent demand. In this study, a new type of sediment microbial fuel cell coupled floating bed (FB-SMFC) was developed to repair eutrophic water and sediment in a cleaner way. The effect of electrode spacing on the power generation capacity and the synchronous remediation of pollutants from eutrophic water and sediment were studied. When the electrode distance was 60 cm, the maximum power generation and pollutant removal effects were obtained. At the end of the experiment, the maximum output voltage was 0.4 V, and the chemical oxygen demand (COD_Cr_, potassium dichromate method), total nitrogen (TN), and total phosphorus (TP) contents in the overlying water were 8 mg/L, 0.7 mg/L, and 0.39 mg/L. The corresponding removal rates were 88.2%, 78.8%, and 59.0%, respectively. The removal rates of organic matter and TN in the sediment were 12.8% and 86.4%, respectively, and the fixation rate of TP was 29.2%. Proteobacteria was the dominant phylum of bacteria in the sediment and anode. Many anaerobic bacteria were found in the overlying water, which facilitated denitrification. Overall, the results of this research revealed a highly efficient and reliable strategy for eutrophic water and sediment remediation, aquatic ecosystems restoration, and human health protection.

## 1. Introduction

Energy and environmental crises have become severe challenges and are arousing worldwide concerns today. Anthropogenic eutrophication poses a threat to the environment and the economy, as well as to human health [1,2]. The recent climate change agreement in Paris highlights the importance of aggressively decarbonizing the energy economy and developing new technologies, especially the generation of environmentally clean electrical energy [3]. Microbial fuel cells (MFCs), as a new type of energy and environmental treatment technology, convert the chemical energy of organic matter in wastewater into electricity, which not only purifies sewage but also generates energy [4,5]. The sediment microbial fuel cell (SMFC) is greatly simplified compared to the MFC. Its single-chamber and membrane-less structure reduces its cost while increasing its power generation capacity, which makes it more suitable for practical applications and increases its development value [6,7]. In the SMFCs, the mass transfer of organic matter at the anode is the key limitation to the power generation capacity and durability [8,9,10].

In plants, most of the organic matter produced by photosynthesis is eventually secreted by the root system of the plant into the rhizosphere environment. The root of the plant is conducive to the growth and attachment of microorganisms [11]. Based on this feature of plants, researchers have added plants to SMFCs for in situ collaborative power generation [12]. Plant-sediment microbial fuel cells (P-SMFCs) can utilize organic matter secreted by plant roots. Their power density output can be seven times higher than that of SMFCs without plants [13]. Moreover, some studies have found that plant microbial fuel cells (PMFCs) can also absorb heavy metals [14].

To date, the applications of PMFCs have extended to the fields of ecological engineering systems, artificial wetland microbial fuel cell systems, and moss microbial fuel cell systems [15,16]. The performance of PMFCs is influenced by the type of bioreactor, external resistance, hydraulic retention time (HRT), plant species and health, electrode materials and type, distance between electrodes, and positioning of electrodes [16]. Vascular plants, macrophytes (hydrophytes), and wetland or marshy land grasses are used in PMFCs [17,18,19,20,21]. Mohan et al. reported that a hybrid system could effectively treat wastewater when they treated domestic sewage and fermented distillery effluents using a miniature floating macrophyte ecosystem (FME) in a SMFC [22]. Bioelectricity was obtained during wastewater treatment using a SMFC combined with a submerged and emergent macrophyte-based system (SEMS) [23]. In addition, PMFCs can be incorporated into agricultural land, wetlands, and wastelands that are not suitable for food production to obtain bioenergy without affecting food production [16,24]. Duckweed-microbial fuel cell reactors removed 71% of the boron from wastewater [25]. A P-SMFC system was constructed by combining calamus with a SMFC. Within 367 days, the degradation rate of pyrene and benzopyrene in shallow lake sediments increased by at least 70% compared to a SMFC without plants [26]. PMFCs have been used in sediment and surface water remediation through the degradation of organic matter and rhizodeposits in sediments [27,28]. Xu et al. built a SMFC to assess the removal of NO_3_^−^ from sediment and surface water and found that the coexistence of electrogenesis and plants increased the nitrate removal rate by 23.1% compared to the control SMFC [29].

The electrode gap has an important effect on the performance of microbial fuel cells. As the electrode gap increases, the distance that protons produced by electricity-producing microorganisms need to travel to the cathode increases, thereby increasing the resistance of the reaction system [30,31]. At the same time, if the electrode spacing is too small, a small amount of O_2_ in the air will penetrate the anode and react with the electricity-producing microorganisms. This consumes some of the electrons and results in a reduction of the output current and an increase in the internal resistance [32,33]. In an experiment with a microbial fuel cell coupled to an artificial wetland, it was found that when the electrode gap was set to 3.3 cm, 6.6 cm, 13.2 cm, and 19.8 cm, the maximum power density of 0.08 W/m^2^ was achieved at 6.6 cm [34]. The performance of a single-chamber microbial fuel cell with a low-cost polypropylene separator was studied, and it was shown that as the electrode gap increased from 0 mm to 9 mm, the maximum power density of 488 mW/m^2^ was obtained in the polarization analysis at 6 mm [35].

The existing research on the electrode gap has mainly focused on small-scale MFCs in which the distance between the electrodes is relatively small. The setup in our study is a SMFC coupled floating bed system (FB-SMFC), for which there have been few studies on the influence of the electrode distance. This study focuses on (1) investigating the effects of electrode distance on the bioelectricity generation efficiency in FB-SMFCs, (2) evaluating the pollutant removal in water and sediments by FB-SMFCs under different electrode distances, and (3) exploring the microbial community structure and the mechanism of bioelectricity generation and pollutant removal in FB-SMFCs. A novel FB-SMFC technique is proposed for the generation of bioelectricity and synchronous in situ remediation of eutrophic water and sediment.

## 2. Materials and Methods

### 2.1. Material Preparation

Synthetic wastewater prepared using analytically pure C_6_H_12_O_6_, NH_4_Cl, NaHCO_3_, KNO_3_, NaNO_2_, KH_2_PO_4_, K_2_HPO_4_, CaCl_2_·2H_2_O, and MgSO_4_·7H_2_O was used as the influent. The water quality of the artificial wastewater is listed in Table 1.

The sediment was collected from the landscape lake of Tianjin University (N: 38°59′49′′; E: 117°18′28′′). Based on the results of preliminary tests, calamus was selected as the test plant because of its good water quality purification effect. The anode electrode materials were carbon felt (Beijing Jinglong Carbon Technology Co., Ltd., Beijing, China) and graphene (Chengdu Institute of Organic Chemistry, Chinese Academic of Science, Chengdu, China). The electrodes were pretreated with acetone, HCl, and NaOH to remove the impurities on them. The graphite and carbon felt electrodes were modified with a 1 g/L graphene dispersion. The cathode electrode consisted of a 10-mesh stainless-steel mesh with the area of 26 cm^2^ and 1 kg of activated carbon particles (Zhengzhou Huanyu Technology Co., Ltd., Zhengzhou, China) with the diameter of 3 mm and length of 2~4 cm.

### 2.2. Lab-Scale FB-SMFCs

**Figure 1:**
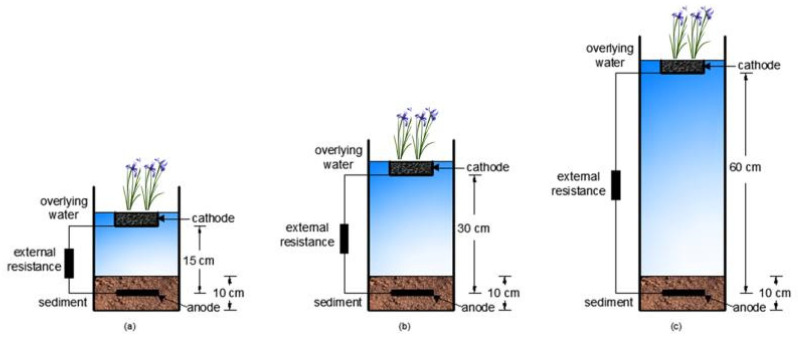
Schematic diagram of (**a**) P-SMFC1, (**b**) P-SMFC2, and (**c**) P-SMFC3.

Three parallel laboratory-scale FB-SMFCs (Figure 1) were developed and named P-SMFC1, P-SMFC2, and P-SMFC3. A glass box was used as the experimental container (25 cm diameter and 100 cm height). The sediment depth was 10 cm. The electrode distances of P-SMFC1, P-SMFC2, and P-SMFC3 were 15 cm, 30 cm, and 60 cm, respectively. Calamus was used as the plant, and the anode electrodes were graphene-modified carbon felt with an area of 9 cm × 9 cm. The cathode electrode was composed of activated carbon particles and a stainless-steel mesh and connected to the anode with an external resistor by wires. The resistance of the resistor was 1000 Ω. During the experiment, no nutrients were added except for the regular addition of tap water without chlorine to replace the evaporated water. The power generation performance of the three FB-SMFCs and the removal rate of pollutants in the sediment and overlying water were studied. The three FB-SMFCs were placed at room temperature (approximately 20 °C). The light intensity was 2700 lx, and the light and dark periods were 10 h and 14 h, respectively.

### 2.3. Sampling and Analysis

After successful setup and operation for one month, the indices were measured from September 2019 to October 2019. The voltages were measured using a multi-channel data logger (provided by Shenzhen Neware Electronics Co., Ltd., Shenzhen, China) and recorded by a computer at intervals of 1 min. Water samples were collected at approximately 5–10 cm below the surface of water every three days from the FB-SMFC systems using syringes with 0.45 mm microporous filter membranes. The chemical oxygen demand (COD_Cr_), total nitrogen (TN), total phosphorus (TP), and ammonium nitrogen (NH_4_^+^-N) were analyzed immediately in the laboratory using a Digital Reactor Block 200 and a HACH DR 2800 spectrophotometer according to the standard method prescribed by HACH Company, USA. At the beginning and end of the experiment, sediment samples were taken from the sludge near the anode of the battery (2–3 cm). Organic matter was measured in accordance with the monitoring methods for municipal sewage treatment of plant sludges (Ministry of Housing and Urban-Rural Development of the People’s Republic of China). Triplicate samples were analyzed for each sample.

At the end of the experiment, a piece of 2 × 2 cm^2^ graphite felt in the central area of the anode and 5 g of activated carbon at the main root of the cathode plant were removed for bacterial diversity analysis. The bacterial diversity was analyzed using Illumina MiSeq sequencing at Biomarker Technologies (Beijing, China).

## 3. Results and Discussion

### 3.1. Electricity Generation Performance of FB-SMFCs

**Figure 2:**
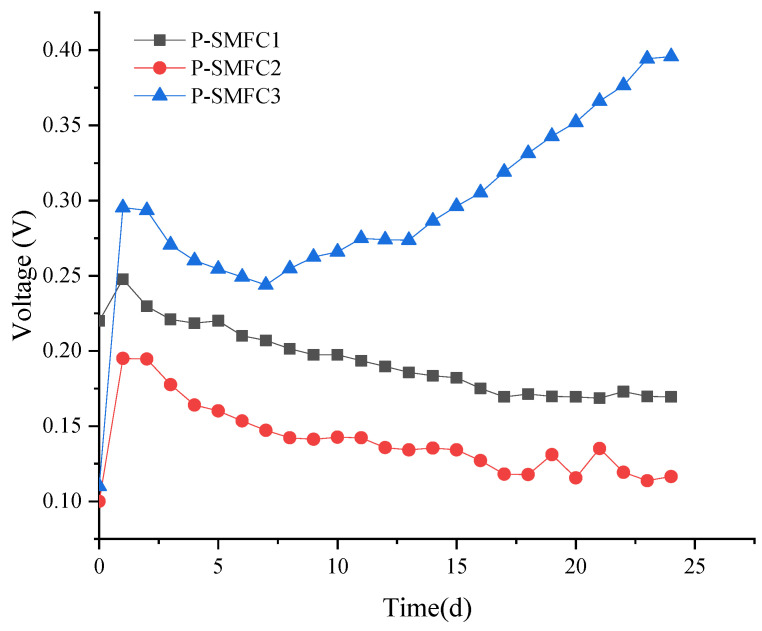
Output voltage of the three FB-SMFCs.

Figure 2 shows the voltage outputs of the three FB-SMFCs. After the systems were started, P-SMFC1, P-SMFC2, and P-SMFC3 gained the initial voltages of 0.22 V, 0.1 V, and 0.11 V immediately. This initial voltage resulted from the chemical and biological reactions caused by the potential difference between the cathode and anode [36]. The voltage rapidly increased and SMFC reached 0.25 V, 0.2 V, and 0.3 V after one day of operation. Subsequently, the voltages of P-SMFC1 and P-SMFC2 gradually decreased. This is possibly because of gradual enrichment of electricity-producing bacteria on the anode of the cell, the output voltage of the battery gradually increased, and as the organic matter in the overlying water and sediment gradually decreased, the cell voltage showed a downward trend. The voltage of P-SMFC3 decreased at first, then gradually increased and reached its maximum (0.4 V) at the end of the experiment. Sajana et al. reported that power production reduced with a decrease in pH but increased with a decrease in external resistance and distance between the electrodes [31].

The results showed that power density increased with decreasing electrode spacing. The ohmic losses in the SMFC depend on the electrode spacing. When the distance between the electrodes is reduced, the protons have less distance to travel. This reduces the ohmic resistance during power generation. Liu et al. reported that the power increased by approximately 60% when the electrode spacing was decreased from 4 to 2 cm in a single-chamber MFC owing to the decrease in internal resistance [37]. Hong et al. reported that when the electrode spacing was decreased from 100 cm to 12 cm, the maximum power density increased from 0.37 to 1.01 mW/m^2^ [9].

### 3.2. Pollutant Removal Effect in Overlying Water

#### 3.2.1. COD_Cr_ Removal Effect in Overlying Water

**Figure 3:**
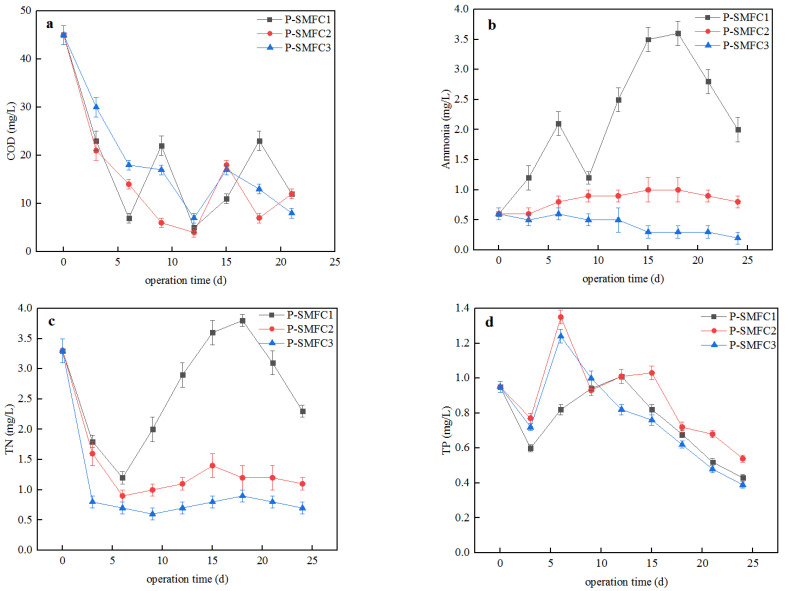
Changes in COD_Cr_ (**a**), NH4^+^-N (**b**), TN (**c**), and TP (**d**) concentrations over time in overlying water in the three setups.

Figure 3a shows the change in the COD_Cr_ in the three FB-SMFCs. As can be seen from the figure, the COD_Cr_ decreased rapidly in the first five days. This may be caused by the adsorption of organic matter in water by plants and sediment in the early stage of the experiment. On the fifth day, the COD_Cr_ in P-SMFC1, P-SMFC2, and P-SMFC3 decreased to 7, 14, and 18 mg/L, respectively. The best COD_Cr_ removal efficiency of 84.4% was obtained in P-SMFC1, which may be related to the smaller volume of overlying water in P-SMFC1. This removal rate was close to the COD_Cr_ removal rate obtained by Doherty et al., while the COD_Cr_ concentration of the effluent in this study was much lower [38]. Wang et al. reported that the COD_Cr_ removal efficiency reached 97.63% when the electrode spacing was 30 cm [39]. The COD_Cr_ concentration fluctuated greatly from days 5 to 20, but all the COD_Cr_ concentrations were lower than 23 mg/L. It can be attributed to the release of a small amount of organic matter adsorbed by the sediments and plants in the early stage, while most of them were utilized by the electrogenic bacteria at the anode. The removal efficiencies of COD_Cr_ in the three FB-SMFCs were 73.3%, 73.3%, and 82.2%, respectively, and the best removal efficiency was achieved when the electrode spacing was 60 cm (P-SMFC3). Overall, the removal efficiency of COD_Cr_ in the overlying water of the three systems had approximately the same values during the entire experimental period. The results indicate that the electrode distance had little effect on the removal rate of COD_Cr_ in the overlying water. The electrode distance of 60 cm in P-SMFC3 indicates that the FB-SMFC could achieve good COD_Cr_ removal efficiency even under the condition of a large electrode distance.

#### 3.2.2. Nitrogen Removal Effect in Overlying Water

Nitrogen is one of the basic nutrients for plant growth, but excessive nitrogen can lead to toxic algal blooms and eutrophication of aquatic ecosystems through oxygen depletion in the water. Figure 3b shows the change in the NH_4_^+^-N concentration in the overlying water of the FB-SMFCs. The NH_4_^+^-N concentration of P-SMFC1 fluctuated greatly. The concentration of NH_4_^+^-N increased in the first 6 days and reached 2.1 mg/L, and then decreased to 1.2 mg/L in the following 3 days. The concentration of NH_4_^+^-N subsequently increased continuously and reached 3.6 mg/L on the 18th day, and then gradually decreased to 2.0 mg/L on the 24th day. The concentration of NH_4_^+^-N increased slowly in the overlying water of the P-SMFC2 system from the initial concentration of 0.6 mg/L to 1 mg/L in the first 18 days and decreased slightly to 0.8 mg/L at the end of the experiment. The concentration of NH_4_^+^-N in P-SMFC3 was nearly unchanged in the first 12 days and then decreased slightly from 0.5 mg/L to 0.2 mg/L at the end of the experiment. It can be seen that the increasing trend of NH_4_^+^-N concentration was more obvious and the fluctuation was greater as the electrode distance decreased because NH_4_^+^-N was released from the sediment and the small electrode distance made it easy for overlying water to be affected by sediment. The results show that the best removal efficiency of NH_4_^+^-N was achieved in P-SMFC3 (66.7%) at the electrode distance of 60 cm. Aquatic plants are thought to significantly affect the nitrogen cycle in water systems through the effective absorption of sediments and dissolved nitrogen in water through their roots, the release of oxygen and organic matter, and the creation of anaerobic microenvironments in the rhizosphere which facilitate nitrogen removal by improving the nitrification and denitrification processes. The plants in the FB-SMFCs supply oxygen to the aerobic cathode and produce rhizosphere oxygen and rhizosphere precipitation. The large surface area of the plant roots and the complex aerobic-anaerobic environment promote the growth and attachment of microorganisms.

The mechanisms for the removal of TN in the overlying water include volatilization, nitrification/denitrification, plant uptake, and substrate adsorption. Moreover, SMFCNO_2_^−^ and NO_3_^−^ can act as electron receptors that promote nitrogen removal at the cathodes of FB-SMFCs. Virdis et al. reported that nitrite and/or nitrate can act as electron receptors in the cathode of SMFCs, thereby reducing the content of nitrite and nitrate in the overlying water [40]. The removal rate of TN in the overlying water is affected by the pH, electrode distance, and external resistance [31]. The changes in TN in the three FB-SMFCs are shown in Figure 3c. The concentration of TN decreased rapidly during the first six days. P-SMFC3 had the best TN removal rate (78.8%) in which the concentration of TN was reduced to 0.7 mg/L. The concentration of TN in the three systems tended to increase after the first six days, which may have been caused by the release of nitrogen from the sediments to the overlying water. Excessive oxygen in the overlying water decreases the denitrification efficiency significantly, but the denitrification rate increases with the organic matter concentration in the sediments [15]. The concentration of TN in P-SMFC1 increased continuously from 1.2 mg/L to 3.8 mg/L over the following 12 days and then began to decrease. At the 24th day, the concentration of TN in P-SMFC1 was 2.2 mg/L, and those in P-SMFC2 and P-SMFC3 were 1.1 mg/L and 0.7 mg/L, respectively. The nitrogen removal efficiencies of the three FB-SMFCs were 30.3%, 66.7%, and 78.8%, respectively, and the best removal efficiency was achieved when the electrode space was 60 cm (P-SMFC3). Wang et al. reported that the best removal efficiencies of organic matter and nitrogen were achieved simultaneously when the electrode spacing was 30 cm [39]. The maximum removal rate of nitrate nitrogen was achieved when the electrode gap was 45 cm [41]. Sajana et al. reported that the total nitrogen removal rate in overlying water increases with the pH and electrode spacing and decreases with increasing external resistance [31].

#### 3.2.3. Phosphorus Removal Effect in Overlying Water

Figure 3d shows the change in TP concentration in the overlying water of the three FB-SMFCs with the running time. Between days 0 and 6, the TP concentration first decreased and then increased. After the sixth day, the TP concentration in P-SMFC2 and P-SMFC3 decreased, while the concentration in P-SMFC1 continued to increase. Between days 9 and 12, the TP concentration in the overlying water of the FB-SMFCs showed a decreasing trend with an increase in the system operating time. The fluctuation of TP concentration was also related to the adsorption and release of sediment. P-SMFC3 generated the largest voltage (Figure 2) and had the best TP removal effect in the overlying water. On the 24th day, the TP concentrations in the overlying water of P-SMFC1, P-SMFC2, and P-SMFC3 were 0.43 mg/L, 0.54, and 0.39 mg/L, respectively. This result is consistent with the power generation results of the three systems, in which the electromigration of phosphate is the main mechanism of TP removal in the overlying water [42,43]. The removal efficiencies of TP in the three FB-SMFCs were 54.7%, 43.2%, and 59.0%, respectively, and the best removal efficiency was achieved when the electrode spacing was 60 cm (P-SMFC3). The removal rate of TP in P-SMFC3 was lower than the 95.06% in a CW-MFC system with the electrode distance of 17.5 cm constructed by Xu et al. [44].

### 3.3. Analysis of Pollutant Removal Effect in Sediments

**Figure 4:**
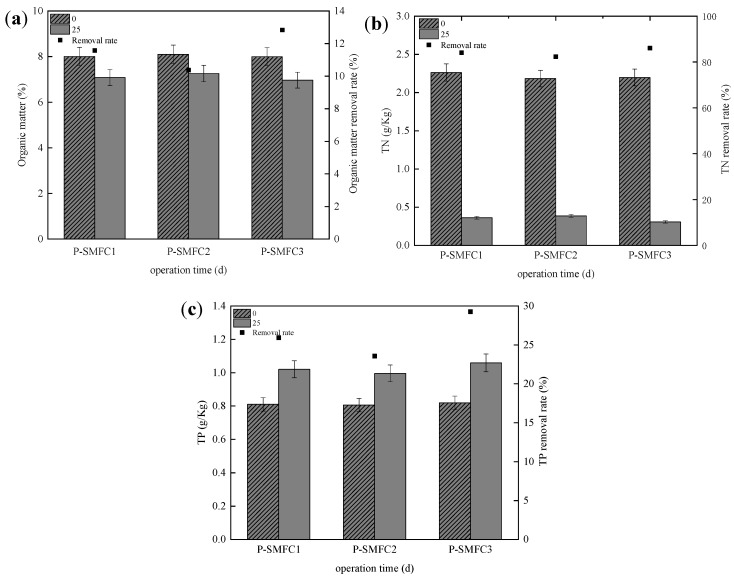
Amounts and removal rates of organic matter (**a**), TN (**b**), and TP (**c**).

The changes in organic matter in the sediments of the three FB-SMFCs are shown in Figure 4a. The removal rates of organic matter in P-SMFC1, P-SMFC2, and P-SMFC3 reached 11.5%, 10.3%, and 12.8%, respectively. The organic matter removal efficiency increased with the battery voltage [45]. P-SMFC3 achieved the best organic removal effect and the highest output voltage.

Nitrogen in polluted river sediments is a significant problem because it leads to eutrophication and is discharged into surface water continuously [46]. The changes in TN in the sediments of the three experimental systems are shown in Figure 4b. All three FB-SMFCs had good sediment TN removal efficiency. The removal rates of P-SMFC1, P-SMFC2, and P-SMFC3 were 84.1%, 82.3%, and 86.4%, respectively. P-SMFC3 achieved the best TN removal effect. This can be related to the larger voltage generated in P-SMFC3, which promoted NH_4_^+^ migration into the surface water. In all respects, the performance of P-SMFC3 was the best. The result may be because the anaerobic environment of the anode would be greatly affected by the oxygen in the overlying water when the distance between electrodes was less than 60, resulting in poor system performance.

Nitrogen release from sediments is an important source of pollution in the overlying water in aquatic ecosystems. The migration and transformation of nitrogen in sediments may be due to electricity generation by NH_4_^+^ acting as an electron donor for the anode [43] or the synthesis of organic compounds by bacterial nitrification of NH_4_^+^, which is used by heterotrophs for power generation. The release of NH_4_^+^ from pore water to the overlying water under the action of electromigration and mineralization of organic nitrogen can also cause the release of nitrogen in sediments [47]. Some microbes that exist in sediments, such as *Thiobacillus* and *Sulfurovum*, can participate in denitrification and reduce nitrates by acting as electron receptors to N_2_ [48].

The change in the total phosphorus in the sediment is shown in Figure 4c. At the end of the experiment, the concentration of TP near the anode in the three SMFC coupled floating bed systems increased significantly. The TP fixation rates of P-SMFC1, P-SMFC2, and P-SMFC3 increased by 25.9%, 23.5%, and 29.2% compared to their respective rates on the first day, and P-SMFC3 had the highest TP fixation rates. Comparing Figure 2, Figure 3d and Figure 4c, it can be seen that the TP fixation rate in the sediment of the three setups corresponds to the removal of TP in the overlying water and the output voltage of the battery. This is because the potential difference between the electrodes promotes the migration of phosphate ions into the sediment. At the same time, the anode, acting as an electron acceptor, promoted the stabilization of phosphate-adsorbed metal oxides in the sediment, thereby inhibiting the release of phosphorus into the overlying water [43].

### 3.4. Diversity Analysis of Bacteria

#### 3.4.1. Microbial Diversity Index Analysis of Bacteria on Sediment, Anode, and Cathode

The effect of electrode distance on the diversity and functional genes of microbial communities and the effect of microorganisms on contaminants during the process of microbial stabilization were explored using high-throughput sequencing of the 16S rRNA gene. The Goods coverage indices confirmed that this sequencing result is a good summary of the actual situation in the samples. The Chao1 and ACE indices reflect the community richness, that is, the number of species in the community, while the Shannon and Simpson indices represent the community diversity and comprehensively reflect the richness and evenness of the species in the community.

The sediments, anodes, and cathodes of P-SMFC1, P-SMFC2, and P-SMFC3 were sampled, and the bacterial diversity at the end of the experiment was analyzed. The sediments of P-SMFC1, P-SMFC2, and P-SMFC3 are labeled as S1, S2, and S3, their anodes as A1, A2, and A3, and their cathodes as C1, C2, and C3, respectively.

Table 2 shows the alpha diversity index of bacteria in the samples at the end of the experiment. Both the anode and cathode of P-SMFC3 had the highest ACE and Chao 1 indices, followed by P-SMFC2, while those of P-SMFC1 had the lowest indices. Chao index is an index that estimates the number of OTUs contained in a sample. The largest Chao index indicated that P-SMFC3 had the largest species richness, which means that many microorganisms were enriched in P-SMFC3, which improved the efficiency of P-SMFC3′s electron generation. This can also explain to a certain extent the phenomenon that P-SMFC3 obtained the maximum output voltage. In the sediment samples, S2 had the lowest Shannon index and S1 the highest index, while in the anode sample, A3 had the lowest Shannon index and A2 the highest. Corresponding to these results, in the sediment samples, S1 had the lowest Simpson index and S2 the highest, while in the anode samples, A2 had the lowest Simpson index and A3 the highest. The P-SMFC3 anode had the lowest species diversity, which may be caused by the accumulation of electricity-producing microorganisms by the battery. Moreover, comparing the sediment and anode, the species diversity on the anode was smaller than that of the sediment. This is because after P-SMFC had run for 25 days, the anode was enriched with generating microorganisms, thereby reducing the diversity of the anode bacterial community. The Shannon indices of the cathodes of P-SMFC1, P-SMFC2, and P-SMFC3 indicate that among the cathode samples, C3 had the largest species diversity followed by C2, and C1 had the lowest species diversity. The community coverage of each sample exceeded 99.8%. In summary, P-SMFC3 had the largest species richness but the lowest species diversity, which may have been caused by the enrichment of electricity-producing microorganisms in the microbial fuel cells.

**Figure 5:**
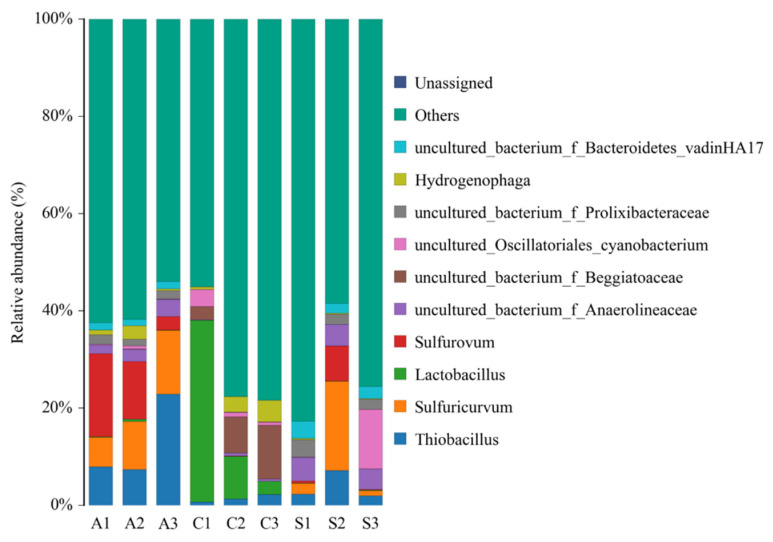
Bacterial community composition of sediment and anode samples at genus level.

The community composition of the bacteria on the sediment and anode at the genus level is shown in Figure 5. There were obvious bacterial community differences between the samples. Compared with the sediment, the contents of Sulfuricurvum, Thiobacillus, Sulfurovum, and Prolixibacteraceae on the anode were higher. Sulfuricurvum is an anaerobic microorganism belonging to the Epsilonbacteraeota, and Sulfuricurvum can degrade difficult-to-degrade organic matter in the sediment. Thiobacillus can perform denitrification reactions to remove nitrogen from the sediment. Sulfurovum is a chemotrophic bacterium belonging to the genus Epsilonproteobacteria. These bacteria use sulfur and thiosulfate as electron donors, carbon dioxide as a carbon source, and oxygen and nitrate as electron acceptors for metabolism [49,50]. Prolixibacteraceae can also remove nitrogen from the sediments. There are many species and a high proportion of bacteria that can be denitrified on the anode [51], which may be an important reason for the significant decrease in the TN content in the sediment. Among the rest of the genera, Anaerolineaceae uses carbohydrates and protein carbon sources for fermentation and metabolism under anaerobic conditions, and Bacteroidetes is a highly efficient carbohydrate metabolizing microorganism. These bacteria have a strong ability to degrade organic matter, which improves the electricity generation ability of the co-matrix electricity-generating bacteria [52,53,54].

Figure 5 also shows the community composition of the battery cathode samples at the bacterial genus level. The bacterial structure composition differences between different cathodes at the genus level are obvious. Lactobacillus accounted for 37.4% of C1 and only 2.6% of C3. Lactobacillus is a rod- or spherical-shaped Gram-positive bacterium that has a relatively good degradation effect on carbohydrates [55]. The high degradation efficiency of COD_Cr_ in the overlying water in P-SMFC1 may be related to the large number of these bacteria. Beggiatoaceae accounted for 2.7% of the bacterial content in C1 and 11.0% in C3. Beggiatoacea has denitrification abilities and can reduce NO_3_^−^ to N_2_ [56]. The presence of these bacteria helped remove organic matter from the overlying water.

#### 3.4.2. Biomarker Microbes of Bacteria on Sediment, Anode, and Cathode

**Figure 6:**
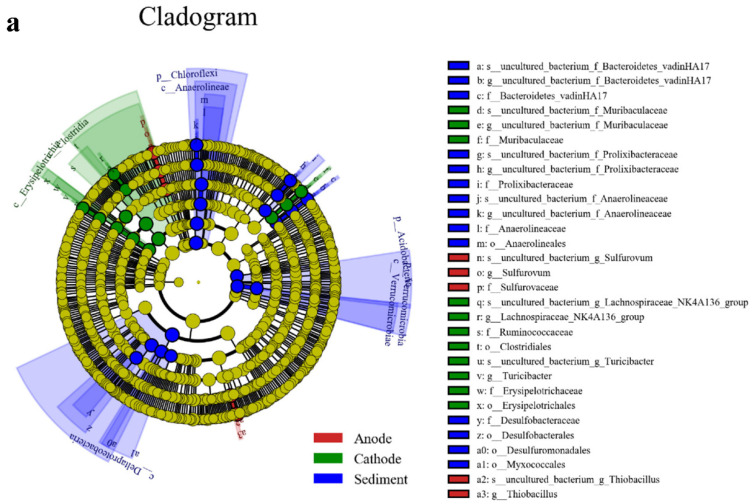

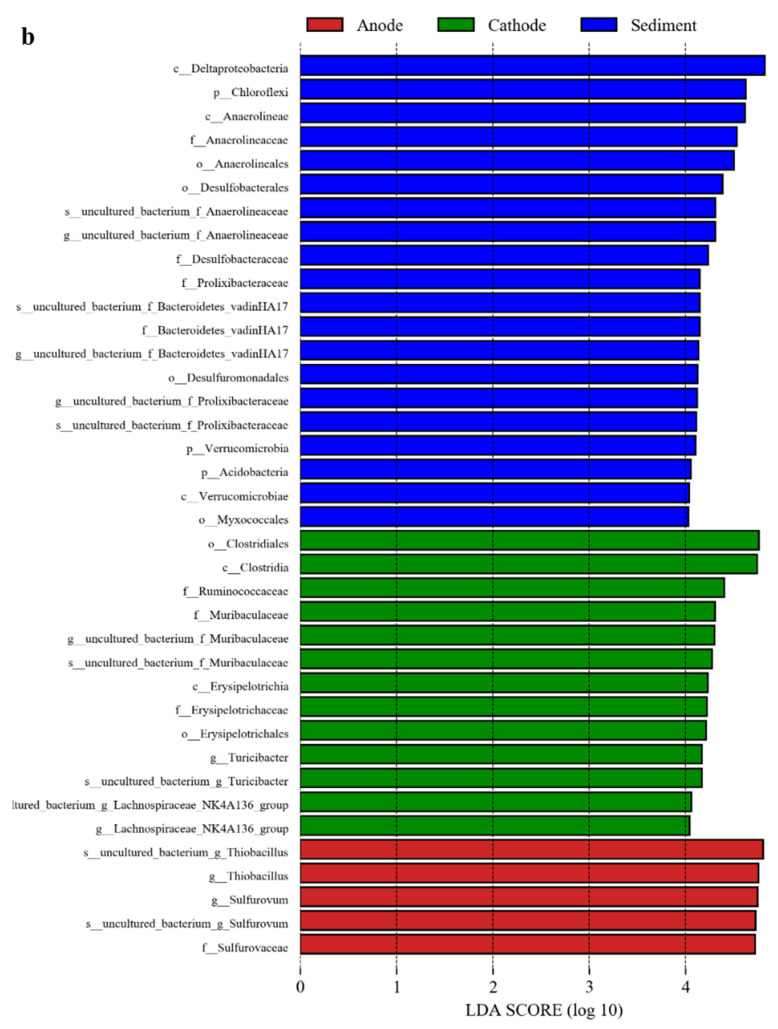
Cladogram based on LEfSe analysis (**a**) and the histogram of the LDA distribution (**b**).

LDA effect size analysis (Lefse) is a biomarker that can be used to compare multiple groups for the identification of species with significant abundance differences between groups. Figure 6 shows the biomarker microbes of the microbial lineages from the phylum to the genus level in the three samples (anode, cathode, and sediment). According to the cladogram of bacteria based on LEfSe analysis (Figure 6a), there were 3, 5, 6, 8, 9, and 7 bacterial species from phylum to species. The histogram of the LDA distribution shows species with LDA scores greater than the set value of 4.0 (Figure 6b). Bacterial phyla such as Lactobacillus, Lactobacillaceae, Bacteroidetes, and Rhodococcus were significantly enriched at the early stage of microbial reduction. At the middle stage of microbial reduction, Oceanospirillales, Halomonas, and Gammaproteobacteria were significantly enriched. Actinobacteria, Micrococcales, and Dietziaceae were significantly enriched at the late stage of microbial reduction. The bacterial genera, which included Thiobacillus, Sulfurovum, and Sulfuricurvum, were significantly enriched on the anode of the P-SMFC systems. In the cathodes of the P-SMFC systems, Clostridiales, Ruminococcaceae, Muribaculaceae, Erysipelotrichales, Turicibacte, and Lachnospiraceae_NK4A136 were significantly enriched. Among them, Clostridiales, Erysipelotrichales, Ruminococcaceae, and Clostridia belong to Firmicutes. Firmicutes was abundant in the cathodes of the P-SMFC systems, which indicates that there was an anaerobic zone in the cathode which could provide an anoxic environment for denitrification on the cathode and facilitate the removal of nitrogen in the overlying water [57]. The proportion of Muribaculaceae, which has a good ability to degrade organic matter [54], in the cathode was more than 2%. Turicibacter was a relatively common dominant bacteria at the genus level [58]. Deltaproteobacteria, Chloroflexi, Anaerolineae, Desulfobacterales, Prolixibacteraceae, Bacteroidetes_vadin HA17, Verrucomicrobia, Acidobacteria, and Myxococcales were significantly enriched in the sediments of the P-SMFC systems. In general, Proteobacteria is the major phylum of Gram-negative bacteria, and many species that belong to Proteobacteria have great potential in degrading organic compounds [59]. Proteobacteria is the dominant bacteria in many MFCs and widely exists in both cathode and anode biofilms [60,61]. Proteobacteria has also been proven to be related to the generation of current in electrochemical systems [62]. Verrrucomicrobia, a bacterial phylum, has considerable ecological impact on nitrogen fixation and methane oxidation and may play an important role in nitrogen fixation in sediments. In addition, Chloroflexi is also present in the sediments and participates in the removal of nitrogen from wastewater [63]. Prolixibacteraceae can also remove nitrogen from the sediments. The results show that there were many species and a high proportion of bacteria that could be denitrified on the anode [51], which may be an important reason for the significant decrease in TN content in the sediments.

#### 3.4.3. Diversity Analysis of Bacteria on Cathode

The alpha diversity indices of the samples were determined, and the species diversity was evaluated using the Shannon index. The Shannon indices of the three groups of samples were 4.195 (C1), 5.2873 (C2), and 5.4809 (C3), respectively. The results show that among the cathode samples, the species diversity of C3 was the highest, followed by C2 and C1.

**Figure 7:**
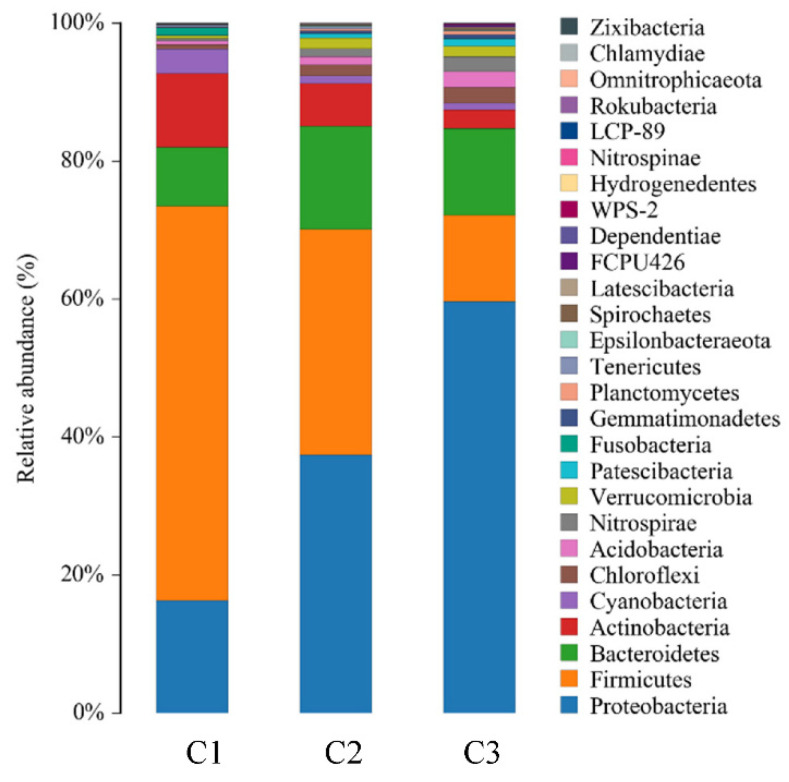
Bacterial community composition of cathode samples at phylum level.

The bacterial community structure of the cathode samples at the phylum level is shown in Figure 7. The most abundant phyla in C3 and C2 was Proteobacteria, which accounted for 59.7% and 37.5% of the phyla, respectively. Proteobacteria is a Gram-negative bacterium, most of which are anaerobic or facultative anaerobic bacteria. Firmicutes was the most abundant phylum in C1 and accounted for 57.2% of the phyla in C1, and for 32.7% and 12.5% of the phyla in C2 and C3, respectively. Firmicutes are Gram-positive bacteria, most of which are anaerobic bacteria. Bacteroidetes accounted for 8.5%, 14.9%, and 12.6% of the bacterial samples in the three cathodes, respectively. C1 also had a high Actinobacteria content of 10.6%. Proteobacteria and Firmicutes were abundant in the cathodes of the P-SMFC systems, which indicates that there was an anaerobic zone in the cathode which could provide an anoxic environment for denitrification on the cathode and facilitate the removal of nitrogen in the overlying water [52,64]. In addition, Nitrospirae was detected at the cathodes of the P-SMFC and accounted for 0.3%, 1.2%, and 2.1% of the bacterial contents in C1, C2, and C3, respectively. Nitrospirae are nitrifying bacteria [65]. The highest proportion of Nitrospirae occurred in P-SMFC3, which indicates that the lower ammonia nitrogen concentration in P-SMFC3 was related to the large number of these bacteria.

The community structure at the genus level shows that the bacterial structure composition was obviously different between the cathodes. Lactobacillus accounted for 37.4% of C1 and only 2.6% of C3. Lactobacillus is a rod-shaped or spherical Gram-positive bacterium that has a relatively good degradation effect on carbohydrates [55]. The good degradation efficiency of COD_Cr_ in the overlying water in P-SMFC1 overlying water may be related to the large number of these bacteria. The proportion of Beggiatoacea in P-SMFC3 was 11.0%, which was higher than that in P-SMFC1 (2.7%) and P-SMFC2 (7.4%). Beggiatoacea is a denitrifying bacterium that can reduce NO_3_^−^ to N_2_ and promote the removal of nitrate from the overlying water [56]. The proportion of Muribaculaceae, which has a good ability to degrade organic matter [54], in the cathode was more than 2%. The presence of these bacteria helped remove organic matter from the overlying water.

## 4. Conclusions

A new type of SMFC coupled floating bed system (FB-SMFC) was constructed. The results showed that the electrode gap had an obvious influence on the performance of the FB-SMFCs. The maximum output voltage (0.4 V) was obtained when the electrode gap was 60 cm, and the best pollutant removal effects in the overlying water and the sediment were achieved. Analysis of the microbial diversity in the electrodes and the sediments confirmed that the FB-SMFCs still had a good enrichment effect on the electricity-generating microorganisms when the electrode gap was large, as well as excellent output voltage and degradation performance. Thus, this study provides a theoretical basis and technical support for in situ remediation of eutrophic water and sediment. The FB-SMFC system provided a strategy for sustainable and renewable energy, aquatic ecosystems restoration, and human health protection.

## Figures and Tables

**Table 1:** Water quality of artificial wastewater. Values are shown as the means ± standard deviations; n = 3.

COD_Cr_ (mg·L^−1^)	Ammonia Nitrogen (mg·L^−1^)	Total Nitrogen (mg·L^−1^)	Total Phosphorus (mg·L^−1^)
90.2 ± 8.1	11.7 ± 5.3	11.9 ± 6.8	2.9 ± 0.3

**Table 2:** Alpha diversity indices of the samples.

Sample ID	ACE	Chao1	Simpson	Shannon	Coverage
A1	1542.645	1554.058	0.0399	5.1732	0.9985
A2	1636.806	1657.962	0.0313	5.3573	0.9986
A3	1677.942	1699.2	0.0639	5.0461	0.998
C1	517.4558	536.12	0.0574	4.1906	0.9986
C2	662.8662	680.2	0.0171	5.2793	0.9982
C3	889.458	917.4688	0.0185	5.4685	0.9984
S1	1692.716	1707.742	0.0036	6.4852	0.9988
S2	1684.148	1693.105	0.0432	5.4107	0.9986
S3	1724.58	1733.456	0.0175	6.1057	0.9988

The alpha diversity index of the samples was counted and the species diversity evaluated using the Shannon index.

## Data Availability

The data can be made available upon request.

## References

[1] Smith V.H., Tilman G.D., Nekola J.C. (1999). Eutrophication: Impacts of excess nutrient inputs on freshwater, marine, and terrestrial ecosystems. Environ. Pollut..

[2] Le Moal M., Gascuel-Odoux C., Ménesguen A., Souchon Y., étrillard C., Levain A., Moatar F., Pannard A., Souchu P., Lefebvre A. (2019). Eutrophication: A new wine in an old bottle?. Sci. Total Environ..

[3] Nowotny J., Dodson J., Fiechter S., Gür T.M., Kennedy B., Macyk W., Bak T., Sigmund W., Yamawaki M., Rahman K.A. (2018). Towards global sustainability: Education on environmentally clean energy technologies. Renew. Sust. Energ. Rev..

[4] Gil G.C., Chang I.S., Kim B.H., Kim M., Kim H.J. (2003). Operational parameters affecting the performance of a mediator-less microbial fuel cell. Biosens. Bioelectron..

[5] Lu N., Zhou S.G., Zhuang L., Zhang J.T., Ni J.R. (2009). Electricity generation from starch processing wastewater using microbial fuel cell technology. Biochem. Eng. J..

[6] Tender L.M., Gray S.A., Groveman E., Lowy D.A., Kauffman P., Melhado J., Tyce R.C., Flynn D., Petrecca R., Dobarro J. (2008). The first demonstration of a microbial fuel cell as a viable power supply: Powering a meteorological buoy. J. Power Sources.

[7] Reimers C.E., Tender L.M., Fertig S., Wang W. (2001). Harvesting energy from the marine sediment-water interface. Environ. Sci. Technol..

[8] He Z., Shao H.B., Angenent L.T. (2007). Increased power production from a sediment microbial fuel cell with a rotating cathode. Biosens. Bioelectron..

[9] Hong S.W., Chang I.S., Choi Y.S., Chung T.H. (2009). Experimental evaluation of influential factors for electricity harvesting from sediment using microbial fuel cell. Bioresour. Technol..

[10] Yuan Y., Zhou S.G., Zhuang L. (2010). A new approach to in situ sediment remediation based on air-cathode microbial fuel cells. J. Soils Sediments.

[11] Hodge A., Berta G., Doussan C., Merchan F., Crespi M. (2009). Plant root growth, architecture and function. Plant Soil.

[12] Han T., Zhao Z.P., Bartlam M., Wang P.P. (2016). Combination of biochar amendment and phytoremediation for hydrocarbon removal in petroleum-contaminated soil. Environ. Sci. Pollut. Res..

[13] De Schamphelair L., Van den Bossche L., Dang H.S., Höfte M., Boon N., Rabaey K., Verstraete V. (2008). Microbial fuel cells generating electricity from rhizodeposits of rice plants. Environ. Sci. Technol..

[14] Guan C.Y., Hu A.Y., Yu C.P. (2019). Stratified chemical and microbial characteristics between anode and cathode after long-term operation of plant microbial fuel cells for remediation of metal contaminated soils. Sci. Total Environ..

[15] Zhang Y.F., Angelidaki I. (2012). Bioelectrode-based approach for enhancing nitrate and nitrite removal and electricity generation from eutrophic lakes. Water Res..

[16] Kabutey F.T., Zhao Q.L., Wei L.L., Ding J., Antwi P., Quashie F.K., Wang W.Y. (2019). An overview of plant microbial fuel cells (PMFCs): Configurations and Applications. Renew. Sust. Energ. Rev..

[17] Nitisoravut R., Regmi R. (2017). Plant microbial fuel cells: A promising Biosystems engineering. Renew. Sust. Energ. Rev..

[18] Maddalwar S., Nayak K.K., Kumar M., Singh L. (2021). Plant microbial fuel cell: Opportunities, challenges, and prospects. Bioresour. Technol..

[19] Yang Y., Zhao Y.Q., Tang C., Xu L., Morgan D., Liu R.B. (2020). Role of macrophyte species in constructed wetland-microbial fuel cell for simultaneous wastewater treatment and bioenergy generation. Chem. Eng. J..

[20] Kothapalli A. (2013). Sediment Microbial Fuel Cell as Sustainable Power Resource. Master’s Thesis.

[21] Li H.N., Qu Y.P., Tian Y., Feng Y.J. (2019). The plant-enhanced bio-cathode: Root exudates and microbial community for nitrogen removal. J. Environ. Sci..

[22] Mohan S.V., Mohanakrishna G., Chiranjeevi P. (2011). Sustainable power generation from floating macrophytes based ecological microenvironment through embedded fuel cells along with simultaneous wastewater treatment. Bioresour. Technol..

[23] Chiranjeevi P., Chandra R., Mohan S.V. (2013). Ecologically engineered submerged and emergent macrophyte based system: An integrated eco-electrogenic design for harnessing power with simultaneous wastewater treatment. Ecol. Eng..

[24] Helder M., Strik D.P.B.T.B., Timmers R.A., Raes S.M.T., Hamelers H.V.M., Buisman C.J.N. (2013). Resilience of roof-top Plant-Microbial Fuel Cells during Dutch winter. Biomass Bioenerg..

[25] Türker O.C. (2018). Simultaneous boron (B) removal and electricity generation from domestic wastewater using duckweed-based wastewater treatment reactors coupled with microbial fuel cell. J. Environ. Manag..

[26] Yan Z.S., Jiang H.L., Cai H.Y., Zhou Y.L., Krumholz L.R. (2015). Complex Interactions Between the Macrophyte Acorus Calamus and Microbial Fuel Cells During Pyrene and Benzo[a]Pyrene Degradation in Sediments. Sci Rep..

[27] Xu L., Wang B.D., Liu X.H., Yu W.Z., Zhao Y.Q. (2018). Maximizing the energy harvest from a microbial fuel cell embedded in a constructed wetland. Appl. Energy.

[28] Liu Y.Y., Zhang H.Q., Lu Z.H., Mendoza M.D., Ma J.X., Cai L.K., Zhang L.H. (2019). Decreasing sulfide in sediment and promoting plant growth by plant-sediment microbial fuel cells with emerged plants. Paddy Water Environ..

[29] Xu P., Xiao E.R., Wu J.M., He F., Zhang Y., Wu Z.B. (2019). Enhanced nitrate reduction in water by a combined bio-electrochemical system of microbial fuel cells and submerged aquatic plant Ceratophyllum demersum. J. Environ. Sci..

[30] Ghangrekar M.M., Shinde V.B. (2007). Performance of membrane-less microbial fuel cell treating wastewater and effect of electrode distance and area on electricity production. Bioresour. Technol..

[31] Sajana T.K., Ghangrekar M.M., Mitra A. (2014). Effect of operating parameters on the performance of sediment microbial fuel cell treating aquaculture water. Aquac. Eng..

[32] Aldrovandi A., Marsili E., Stante L., Paganin P., Tabacchioni S., Giordano A. (2009). Sustainable power production in a membrane-less and mediator-less synthetic wastewater microbial fuel cell. Bioresour. Technol..

[33] Jang J.K., Pham T.H., Chang I.S., Kang K.H., Moon H., Cho K.S., Kim B.H. (2004). Construction and operation of a novel mediator- and membrane-less microbial fuel cell. Process Biochem..

[34] Fang Z., Cheng S.C., Cao X., Wang H., Li X.N. (2017). Effects of electrode gap and wastewater condition on the performance of microbial fuel cell coupled constructed wetland. Environ. Technol..

[35] Kondaveeti S., Moon J.M., Min B. (2017). Optimum spacing between electrodes in an air-cathode single chamber microbial fuel cell with a low-cost polypropylene separator. Bioprocess Biosyst. Eng..

[36] Min B., Kim J.R., Oh S.E., Regan G.M., Logan B.E. (2005). Electricity generation from swine wastewater using microbial fuel cells. Water Res..

[37] Liu H., Cheng S.A., Logan B.E. (2005). Power generation in fed-batch microbial fuel cells as a function of ionic strength, temperature, and reactor configuration. Environ. Sci. Technol..

[38] Doherty L., Zhao X.H., Zhao Y.Q., Wang W.K. (2015). The effects of electrode spacing and flow direction on the performance of microbial fuel cell-constructed wetland. Ecol. Eng..

[39] Wang H.R., Fu B.Y., Xi J.Y., Hu H.Y., Liang P., Huang X., Zhang X.Y. (2019). Remediation of simulated malodorous surface water by columnar air-cathode microbial fuel cells. Sci. Total Environ..

[40] Virdis B., Rabaey K., Yuan Z., Keller J. (2008). Microbial fuel cells for simultaneous carbon and nitrogen removal. Water Res..

[41] Wang L.M., Pang Q.Q., Zhou Y., Peng F.Q., He F., Li W.X., Xu B., Cui Y.B., Zhu X. (2020). Robust nitrate removal and bioenergy generation with elucidating functional microorganisms under carbon constraint in a novel multianode tidal constructed wetland coupled with microbial fuel cell. Bioresour. Technol..

[42] Almatouq A., Babatunde A.O. (2018). Identifying optimized conditions for concurrent electricity production and phosphorus recovery in a mediator-less dual chamber microbial fuel cell. Appl. Energy.

[43] He Z., Kan J.J., Wang Y.B., Huang Y.L., Mansfeld F., Nealson K.H. (2009). Electricity Production Coupled to Ammonium in a Microbial Fuel Cell. Environ. Sci. Technol..

[44] Xu F., Cao F.Q., Kong Q., Zhou L.L., Yuan Q., Zhu Y.J., Wang Q., Du Y.D., Wang Z.D. (2018). Electricity production and evolution of microbial community in the constructed wetland-microbial fuel cell. Chem. Eng. J..

[45] Srivastava P., Yadav A.K., Mishra B.K. (2015). The effects of microbial fuel cell integration into constructed wetland on the performance of constructed wetland. Bioresour. Technol..

[46] Yan H.J., Saito T., Regan J.M. (2012). Nitrogen removal in a single-chamber microbial fuel cell with nitrifying biofilm enriched at the air cathode. Water Res..

[47] Xu P., Xiao E.R., Xu D., Zhou Y., He F., Liu B.Y., Zeng L., Wu Z.B. (2017). Internal nitrogen removal from sediments by the hybrid system of microbial fuel cells and submerged aquatic plants. PLoS ONE.

[48] Huang G.T., Qu L., Ding Y. (2019). Birnessite modified graphite cathode toward efficient autotrophic denitrification of *Thiobacillus denitrificans* in bioelectrochemical system. Desalin. Water Treat..

[49] Haosagul S., Prommeenate P., Hobbs G., Pisutpaisal N. (2020). Sulfide-oxidizing bacteria community in full-scale bioscrubber treating H_2_S in biogas from swine anaerobic digester. Renew. Energy.

[50] Wang Y.J., Singh R.P., Zhang J.Y., Xu Y., Fu D.F. (2019). Nitrogen removal performance of microbial fuel cell enhanced bioretention system. J. Water Supply Res. Technol. Aqua.

[51] Lukwambe B., Yang W., Zheng Y.Q., Nicholaus R., Zhu J.Y., Zheng Z.M. (2018). Bioturbation by the razor clam (*Sinonovacula constricta*) on the microbial community and enzymatic activities in the sediment of an ecological aquaculture wastewater treatment system. Sci. Total Environ..

[52] Cabezas A., Pommerenke B., Boon N., Friedrich M.W. (2015). *Geobacter*, *Anaeromyxobacter* and *Anaerolineae* populations are enriched on anodes of root exudate-driven microbial fuel cells in rice field soil. Environ. Microbiol. Rep..

[53] Liu J., Yi N.K., Wang S., Lu L.J., Huang X.F. (2016). Impact of plant species on spatial distribution of metabolic potential and functional diversity of microbial communities in a constructed wetland treating aquaculture wastewater. Ecol. Eng..

[54] Atasoy M., Eyice O., Cetecioglu Z. (2020). A comprehensive study of volatile fatty acids production from batch reactor to anaerobic sequencing batch reactor by using cheese processing wastewater. Bioresour. Technol..

[55] Vilas Boas J., Oliveira V.B., Marcon L.R.C., Pinto D.P., Simoes M., Pinto A.M.F.R. (2015). Effect of operating and design parameters on the performance of a microbial fuel cell with Lactobacillus pentosus. Biochem. Eng. J..

[56] Schutte C.A., Teske A., MacGregor B.J., Salman-Carvalho V., Lavik G., Hach P., de Beer D. (2018). Filamentous Giant Beggiatoaceae from the Guaymas Basin Are Capable of both Denitrification and Dissimilatory Nitrate Reduction to Ammonium. Appl. Environ. Microbiol..

[57] Wang L., Liu Y.L., Wang C., Zhao X.D., Mahadeva G.D., Wu Y.C., Ma J., Zhao F. (2018). Anoxic biodegradation of triclosan and the removal of its antimicrobial effect in microbial fuel cells. J. Hazard. Mater..

[58] Naphtali P., Mohiuddin M.M., Paschos A., Schellhorn H.E. (2019). Application of high-throughput 16S rRNA sequencing to identify fecal contamination sources and to complement the detection of fecal indicator bacteria in rural groundwater. J. Water Health.

[59] Fang Z., Song H.L., Cang N., Li X.N. (2013). Performance of microbial fuel cell coupled constructed wetland system for decolorization of azo dye and bioelectricity generation. Bioresour. Technol..

[60] Almatouq A., Babatunde A.O., Khajah M., Webster G., Alfodari M. (2020). Microbial community structure of anode electrodes in microbial fuel cells and microbial electrolysis cells. J. Water Process. Eng..

[61] Huang H.B., Cheng S.A., Yang J.W., Li C.C., Sun Y., Cen K.F. (2018). Effect of nitrate on electricity generation in single-chamber air cathode microbial fuel cells. Chem. Eng. J..

[62] Liao C.M., Wu J.L., Zhou L., Li T., Du Q., An J.K., Li N., Wang X. (2018). Optimal set of electrode potential enhances the toxicity response of biocathode to formaldehyde. Sci. Total Environ..

[63] Yuan J.Q., Yuan H.G., Huang S.B., Liu L.J., Fu F.C., Zhang Y.Q., Cheng F.Q., Li J.F. (2021). Comprehensive performance, bacterial community structure of single-chamber microbial fuel cell affected by COD/N ratio and physiological stratifications in cathode biofilm. Bioresour. Technol..

[64] Dunaj S.J., Vallino J.J., Hines M.E., Gay M., Kobyljanec C., Rooney-Varga J.N. (2012). Relationships between Soil Organic Matter, Nutrients, Bacterial Community Structure, and the Performance of Microbial Fuel Cells. Environ. Sci. Technol..

[65] Chen J.F., Zhang L.H., Hu Y.Y., Huang W.T., Niu Z.Y., Sun J. (2017). Bacterial community shift and incurred performance in response to in situ microbial self-assembly graphene and polarity reversion in microbial fuel cell. Bioresour. Technol..

